# A novel deep learning-based model for automated tooth detection and numbering in mixed and permanent dentition in occlusal photographs

**DOI:** 10.1186/s12903-025-05803-y

**Published:** 2025-03-29

**Authors:** Zahra Ghorbani, Seyed Sepehr Mirebeigi-Jamasbi, Mohammad Hassannia Dargah, Mohammad Nahvi, Sara Alsadat Hosseinikhah Manshadi, Zeinab Akbarzadeh Fathabadi

**Affiliations:** 1 Department of Community Oral Health, School of Dentistry, Shahid Beheshti University of Medical Sciences, Tehran, Iran; 2 Research Committee, School of Dentistry, Shahid Beheshti University of Medical Sciences, Daneshju Blvd., Velenjak St., Chamran Highway, Tehran, 1983963113 Iran; 3 Bachelor of science, Electrical engineering, Shahid Beheshti University, Tehran, Iran

**Keywords:** Artificial intelligence, Deep learning, Tooth detection, Tooth numbering, Occlusal photograph, Permanent dentition, Mixed dentition

## Abstract

**Background:**

While artificial intelligence-driven approaches have shown great promise in dental diagnosis and treatment planning, most research focuses on dental radiographs. Only three studies have explored automated tooth numbering in oral photographs, all focusing on permanent dentition. Our study aimed to introduce an automated system for detection and numbering of teeth across mixed and permanent dentitions in occlusal photographs.

**Methods:**

A total of 3215 occlusal view images of maxilla and mandible were included. Five senior dental students, trained under the guidance of an associate professor in dental public health, annotated the dataset. Samples were distributed across the training, validation, and test sets using a ratio of 7:1.5:1.5, respectively. We employed two separate convolutional neural network (CNN) models working in conjunction. The first model detected tooth presence and position, generating bounding boxes, while the second model localized these boxes, conducted classification, and assigned tooth numbers. Python and YOLOv8 were utilized in model development. Overall performance was assessed using sensitivity, precision, and F1 score.

**Results:**

The model demonstrated a sensitivity of 99.89% and an overall precision of 95.72% across all tooth types, with an F1 score of 97.76%. Misclassifications were primarily observed in underrepresented teeth, including primary incisors and permanent third molars. Among primary teeth, maxillary molars showed the highest performance, with precisions above 94%, 100% sensitivities, and F1 scores exceeding 97%. The mandibular primary canine showed the lowest results, with a precision of 88.52% and an F1 score of 93.91%.

**Conclusion:**

Our study advances dental diagnostics by developing a highly precise artificial intelligence model for detecting and numbering primary and permanent teeth on occlusal photographs. The model’s performance, highlights its potential for real-world applications, including tele-dentistry and epidemiological studies in underserved areas. The model could be integrated with other systems to identify dental problems such as caries and orthodontic issues.

## Background

In recent years, the advancement of dentistry has led to the widespread adoption of clinical photography, integrating intraoral images as essential components of patient documentation and treatment planning [1]. Currently, dental photography is widely applied across various fields of dentistry including aesthetic dentistry, orthodontics, oral surgery, periodontology, and implantology [2]. Tooth segmentation and numbering play a vital role in dental care, aiding in the assessment of oral health and the accurate diagnosis of dental issues [3]. However, these tasks are often carried out manually, leading to significant time investment. Moreover, the subjective nature of this process, dependent on the dentist’s judgment, lacks objective standards, potentially compromising the result’s accuracy [4].

Recent years have witnessed a substantial rise in research focusing on the integration of artificial intelligence (AI) in dentistry, specifically in clinical diagnosis and treatment planning. This trend owes much to the remarkable strides made in convolutional neural networks (CNNs), which have completely transformed image analysis by harnessing deep learning techniques [5]. Deep-learning algorithms acquire knowledge from vast datasets rather than adhering to a predefined set of instructions [6]. These systems autonomously extract features from data without the need for initial interpretation by humans [7]. CNNs excel at analyzing intricate images, utilizing mathematical convolution operations to identify local connectivity patterns such as edges and corners [8]. The You Only Look Once (YOLO) algorithm is a real-time object detection method that employs CNNs and a one-stage approach. YOLOv4 has been explored for various applications, including the detection of periodontal bone loss, localization of third molars, and identification of mandibular fractures, achieving an accuracy of around 90% [9].

Numerous studies have highlighted the capabilities of CNN-driven deep-learning techniques in supporting dental clinicians [10]. However, an examination of AI-based studies in dentistry reveals a predominant focus on dental radiographs, with a limited number of studies dedicated to utilizing AI algorithms for diagnosis and treatment planning through dental photographs [1, 5, 11–14]. To the best of our knowledge, among this limited body of research, only three studies have explored tooth numbering, all exclusively focusing on permanent dentition [1, 5, 15].

Early identification and treatment of caries in school-aged children are essential to prevent disease progression, minimize the need for more invasive and costly interventions, and reduce the overall burden of oral diseases [16, 17]. Additionally, health behaviors and habits established during childhood, often persist into adulthood, underscoring the importance of directing epidemiological studies and health promotion efforts towards children at an early age [18]. However, there is currently a lack of a comprehensive model that can be applied during the mixed dentition stage to correctly identify both primary and permanent teeth. Therefore, our study aimed to addresses this gap by developing an automated system using the YOLOv8 technology to detect and number both primary and permanent teeth in occlusal view oral photographs.

## Methods

### Ethical consideration

This study was approved by the ethics committee of Shahid Beheshti University of Medical Sciences (IR.SBMU.DRC.REC.1402.146). At the time of imaging, written informed consent was obtained from the patients or their parents for the future use of their photos for educational or research purposes without revealing the patient’s identity.

### Dataset preparation

Intraoral images were obtained from patients visiting the Orthodontics Department, Dental School, Shahid Beheshti University of Medical Sciences, Tehran, Iran. A single orthodontic imaging operator employed a professional camera to capture images after cleaning all teeth. Images were taken from various angles, including occlusal, frontal, and lateral views, utilizing mirrors for occlusal views. However, only images of the occlusal view were selected for this study. To fulfill this selection, a CNN classifier model trained specifically for this task was employed. The images had already been captured and were stored in the university archives.

To ensure diversity in the dataset, images containing scratches, saliva, and orthodontic brackets were included in the model training. This approach allows the model to be applicable to non-standard images as well. Age limitation was not applied and both pediatric and adult patients were included in the study. Blurry images and redundant images capturing identical tooth surfaces from identical patients were excluded from the study to maintain data integrity.

Annotation of the dataset was conducted by five senior dental students, initially trained for this task under the guidance of an Associate Professor in dental public health. To ensure consistency in labeling, each student annotated twenty photos as a trial and received feedback from the supervisor, addressing any necessary adjustments. The dental students detected and annotated all teeth in the intraoral images by assigning respective tooth numbers using bounding boxes. To facilitate the annotation process and ensure dataset security with remote access for all labelers, a specialized platform was developed. The front-end was built using React JS, while the back-end utilized C#. This platform enabled labelers to create bounding boxes around each tooth, assign labels, and store data, including box coordinates (center, height, and width) and annotated labels, in a structured query language (SQL) server database.

The dataset consisted of intraoral images alongside JavaScript Object Notation (JSON) files, containing data on the spatial positioning of boxes. Additionally, for each photo, a text file was provided, detailing the coordinates, width, and height of each box as a percentage of the original image size, facilitating compatibility with YOLO. Primary teeth comprised a smaller portion of our dataset compared to permanent teeth. Recognizing this imbalance, we made a deliberate adjustment to the standard 8:1:1 dataset split, choosing a 7:1.5:1.5 configuration instead. This adjustment was essential to ensure that the test set included a sufficient number of primary teeth, allowing for a reliable evaluation of our model’s performance. By increasing the proportion of primary teeth in the test set, we aimed to produce meaningful and robust findings and mitigate the impact of any single misclassification, which could otherwise disproportionately affect the precision scores.

### Deep learning model architecture

**Fig. 1:**
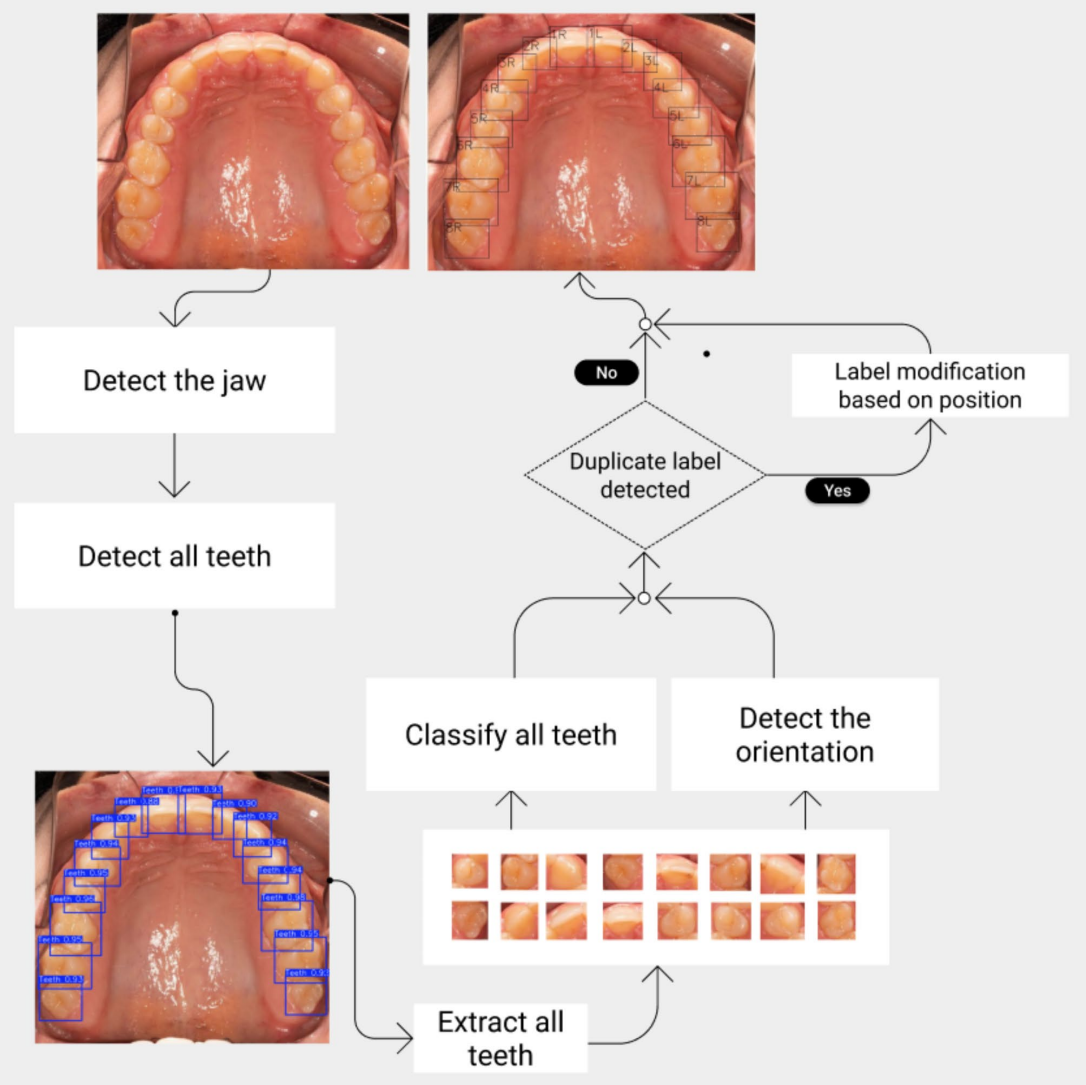
Model architecture of tooth detection and numbering

For this research, we employed two separate CNN models working in conjunction. The first model was dedicated to detecting tooth presence and position, generating bounding boxes, while the second model localized these boxes for all teeth, conducted classification, and assigned tooth numbers. The model was designed to crop, analyze, and number each tooth individually within its bounding box. If two teeth within the same half of the jaw received identical codes, the model then reassessed those teeth together based on their position and assigned new numbers as needed. This approach was particularly necessary when dealing with permanent first and second premolars or deciduous second molars and permanent first molars which may appear similar in certain individuals. Figure 1 displays the workflow of the developed model. Python was utilized in our model development, leveraging widely documented libraries common in the field. For model training, we opted for the PyTorch framework, renowned for its adaptability and effectiveness in deep learning endeavors.

### Model training

Several image augmentations were implemented to enhance model robustness and generalization, including horizontal flip, zoom, crop, photometric distortion, and contrast adjustment. All occlusal images were resized to 640 × 640 pixels for object detection and 128 × 128 pixels for numbering. The dataset was then loaded, and model training commenced.

For object detection, the CNN model was initialized from a pre-trained YOLOv8n model trained on the COCO dataset, and for the tooth numbering, the CNN model was initialized from a pre-trained YOLOv8n-cls model also trained on the COCO dataset. The learning rate of the model was set to 0.0001, and the model underwent an iterative training loop over multiple epochs using batches of 64 images and annotations. Each epoch represented a complete pass through the training dataset, with the training process lasting for 10 epochs for object detection and 40 epochs for tooth numbering. YOLOv8 utilizes a combination of intersection over union (IoU) and generalized IoU (GIoU) loss functions for object detection and vertical federated learning (VFL) for classification.

### Model evaluation

Periodic assessments of the model’s efficacy were conducted using a validation dataset, comparing model predictions against ground-truth annotations to calculate evaluation metrics including sensitivity (recall), mean average precision (mAP), F1 score, which reflects a balance between precision and recall, and the Receiver Operating Characteristic (ROC) curve, which plots the true positive rate against the false positive rate. These metrics offer insights into the model’s accuracy and facilitate monitoring the progress throughout training. Following training, the model underwent testing, and its performance was evaluated using mAP scores with an IoU threshold of 0.5, a common measure for deep learning object detection. The model’s performance was evaluated using the metrics of true positive (TP), false positive (FP), and false negative (FN). TP represents a tooth that is correctly detected and numbered, FP indicates a tooth that is correctly detected but incorrectly numbered, and FN refers to a tooth that is incorrectly detected and numbered. A tooth that is not labeled with any number is an FN. Subsequently, sensitivity (TP/(TP + FN)), precision (TP/(TP + FP)), and the F1 score (2TP / (2TP + FP + FN)) were calculated. We evaluated the model’s potential overfitting by comparing the loss values on the training set with those on the validation set. Substantial discrepancies between training and validation loss values may indicate that the model is performing well on the training data but struggling to generalize to new, unseen data. We employed a confusion matrix to illustrate the model’s frequent classification decisions for each type of tooth. To assess the model’s performance on non-standard images, we conducted a pilot test using 10 photos taken with smartphones and evaluated the model’s performance on them.

### Hardware setup

Data storage was managed on an Ubuntu 22.04 Linux server with two processing cores and 4GB of RAM. Model training was conducted on an Apple M1 system-on-chip (SoC) with 8GB of RAM and a seven-core integrated GPU, running macOS Ventura.

## Results

**Fig. 2:**
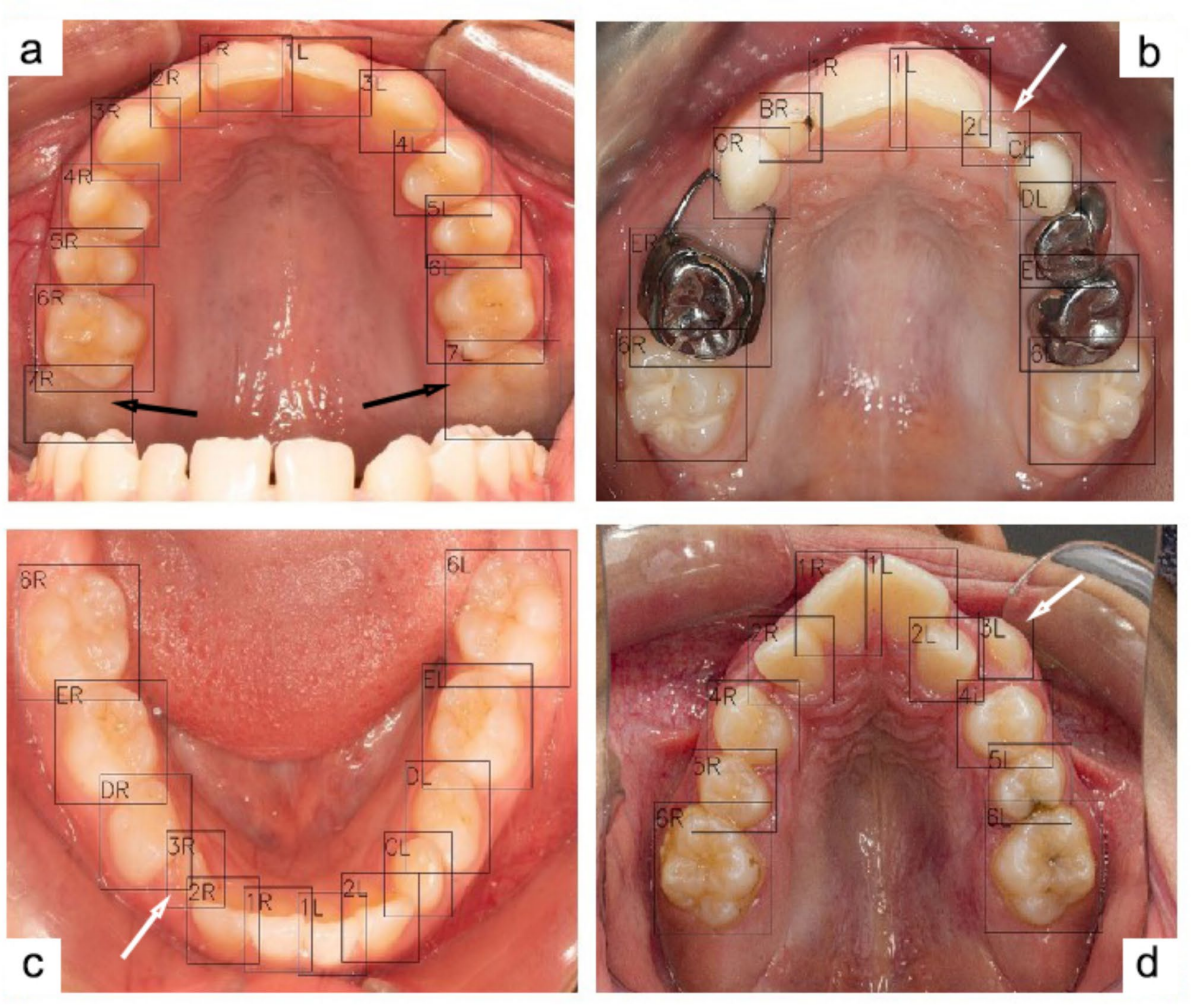
Examples of tooth numbering performed by the model. White arrows indicate partially erupted teeth that were correctly detected and numbered, while black arrows highlight accurate detection and numbering in an obscured area of the mirror

A total of 3215 images, including 1613 maxillary and 1602 mandibular, met the predetermined criteria and were selected for the study. The images were then divided in the 7:1.5:1.5 ratio, resulting in 2251 images in the training group and 482 images each in the validation and testing groups. As reported in Table 1, the model achieved a sensitivity of 99.89% and an overall mAP of 95.72% across all types of teeth. The overall F1 score was 97.76%. Some misclassifications were noted within primary incisors and permanent third molars, which were underrepresented in our dataset. Since these teeth typically do not feature in mixed dentition and were not the primary focus of this study, we excluded them from certain measurements, resulting in slightly improved performance metrics. Figure 2 shows examples of the model performance with perfect precisions.

**Table 1:** Results of the numbering performance in each tooth group

Tooth group	True positive (*n*)	False positive (*n*)	False negative (*n*)	Total teeth (*n*)	Sensitivity (%)	Precision (%)	F1score (%)
All teeth	5720	256	6	5982	99.89	95.72	97.76
All teeth excluding third molar and primary incisors	5656	244	6	5906	99.89	95.86	97.83
Permanent teeth	4785	170	6	4961	99.87	96.57	98.19
Permanent teeth excluding third molar	4738	166	6	4910	99.87	96.61	98.22
All primary teeth	935	86	-	1021	100	91.58	95.60
Primary teeth excluding primary incisors	918	78	-	996	100	92.17	95.92

Upon evaluating the model’s performance for each specific tooth, we observed that the best results were achieved with the permanent central incisors and maxillary second premolars. These teeth demonstrated 100% sensitivity, while precisions and F1 scores exceeded 99%. Among the primary teeth, maxillary molars showed the highest performance, with precisions above 94% and F1 scores surpassing 97%. Excluding third molars and primary incisors, the mandibular primary canine demonstrated the lowest performance, with a precision of 88.52% and an F1 score of 93.91%. Detailed performance metrics for each tooth are provided in Table 2.

**Table 2:** Detailed performance metrics for all teeth

Tooth number	True positive (*n*)	False positive (*n*)	False negative (*n*)	Total teeth (*n*)	Sensitivity (%)	Precision (%)	F1score (%)
Upper 1	460	4	-	464	100	99.14	99.57
Upper 2	398	12	1	411	99.75	97.07	98.39
Upper 3	233	8	1	242	99.57	96.68	98.11
Upper 4	288	7	-	295	100	97.63	98.80
Upper 5	249	2	-	251	100	99.20	99.60
Upper 6	443	20	-	463	100	95.68	97.79
Upper 7	188	5	1	194	99.47	97.41	98.43
Upper 8	25	1	-	26	100	96.15	98.04
Lower 1	492	4	-	496	100	99.19	99.60
Lower 2	476	8	2	486	99.58	98.35	98.96
Lower 3	339	15	1	355	99.71	95.76	97.69
Lower 4	301	13	-	314	100	95.86	97.89
Lower 5	229	10	-	239	100	95.82	97.86
Lower 6	439	39	-	478	100	91.84	95.75
Lower 7	203	19	-	222	100	91.44	95.53
Lower 8	22	3	-	25	100	88	93.62
Upper A	1	2	-	3	100	33.33	50
Upper B	16	6	-	22	100	72.73	84.21
Upper C	162	21	-	183	100	88.52	93.91
Upper D	130	8	-	138	100	94.2	97.01
Upper E	193	11	-	204	100	94.61	97.23
Lower C	96	9	-	105	100	91.43	95.52
Lower D	131	10	-	141	100	92.91	96.32
Lower E	206	19	-	225	100	91.56	95.59

**Fig. 3:**
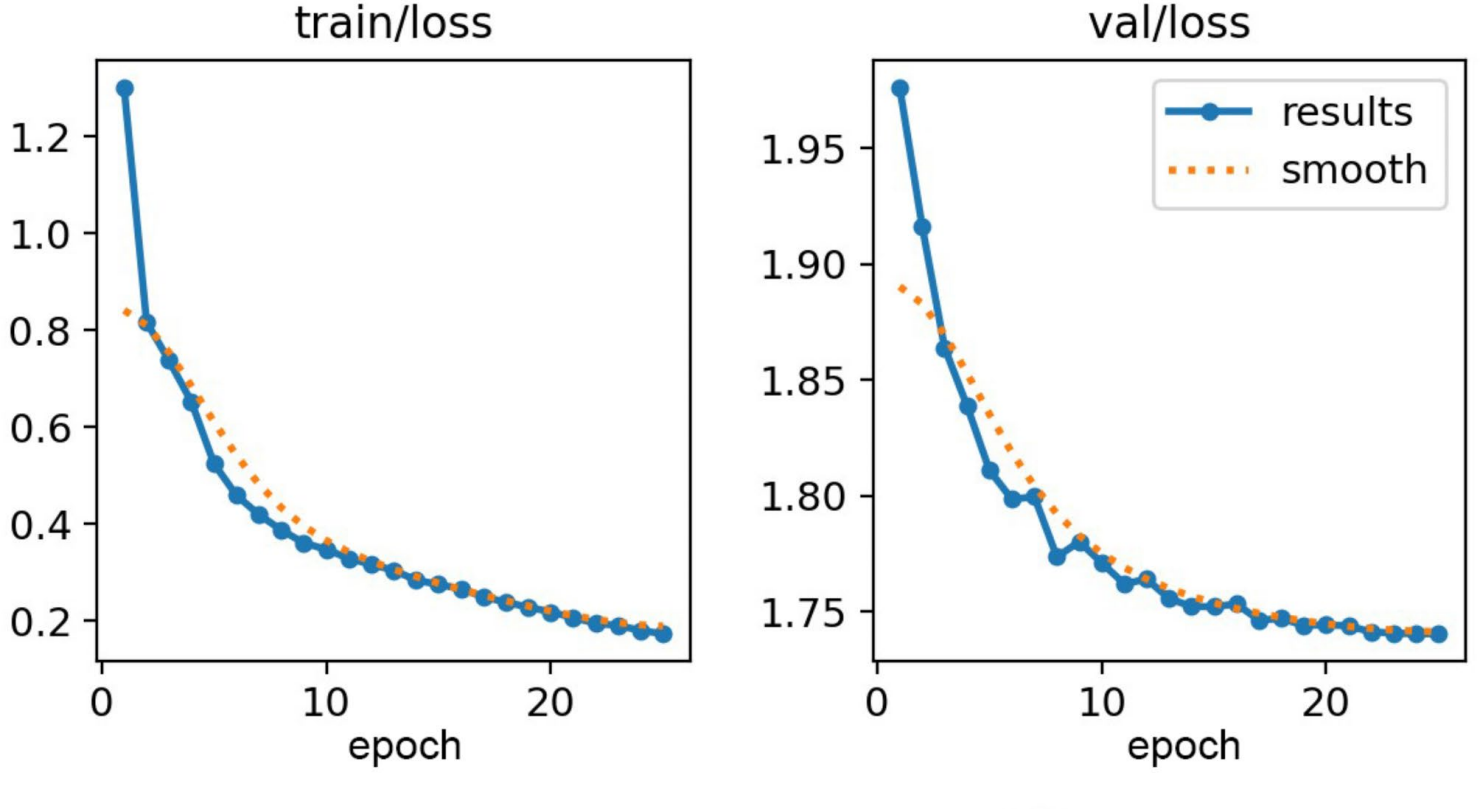
Evolution of loss values for test set and validation set through different epochs

As shown in Fig. 3, our model demonstrated effective learning throughout the training process, with a steady decrease in both training and validation losses over 40 epochs, with only a minor and temporary reduction in the validation curve slope at the beginning. This consistent downward trend in validation loss, without any signs of divergence from the training loss, indicates that our model did not suffer from overfitting during training. The initial change in the slope of the validation loss curve likely reflects the model’s transition from learning basic patterns to capturing more complex features within the dataset. This phase, where the slope temporarily decreases, could indicate a period of adaptation, where the model was refining its understanding of the intricate patterns present in the data. As the model began to generalize more effectively, it moved past this plateau, resuming a steady decline in validation loss. While training continued until 40 epochs, there was minimal change in the loss values after epoch 20, indicating that the model had effectively learned at that point.

**Fig. 4:**
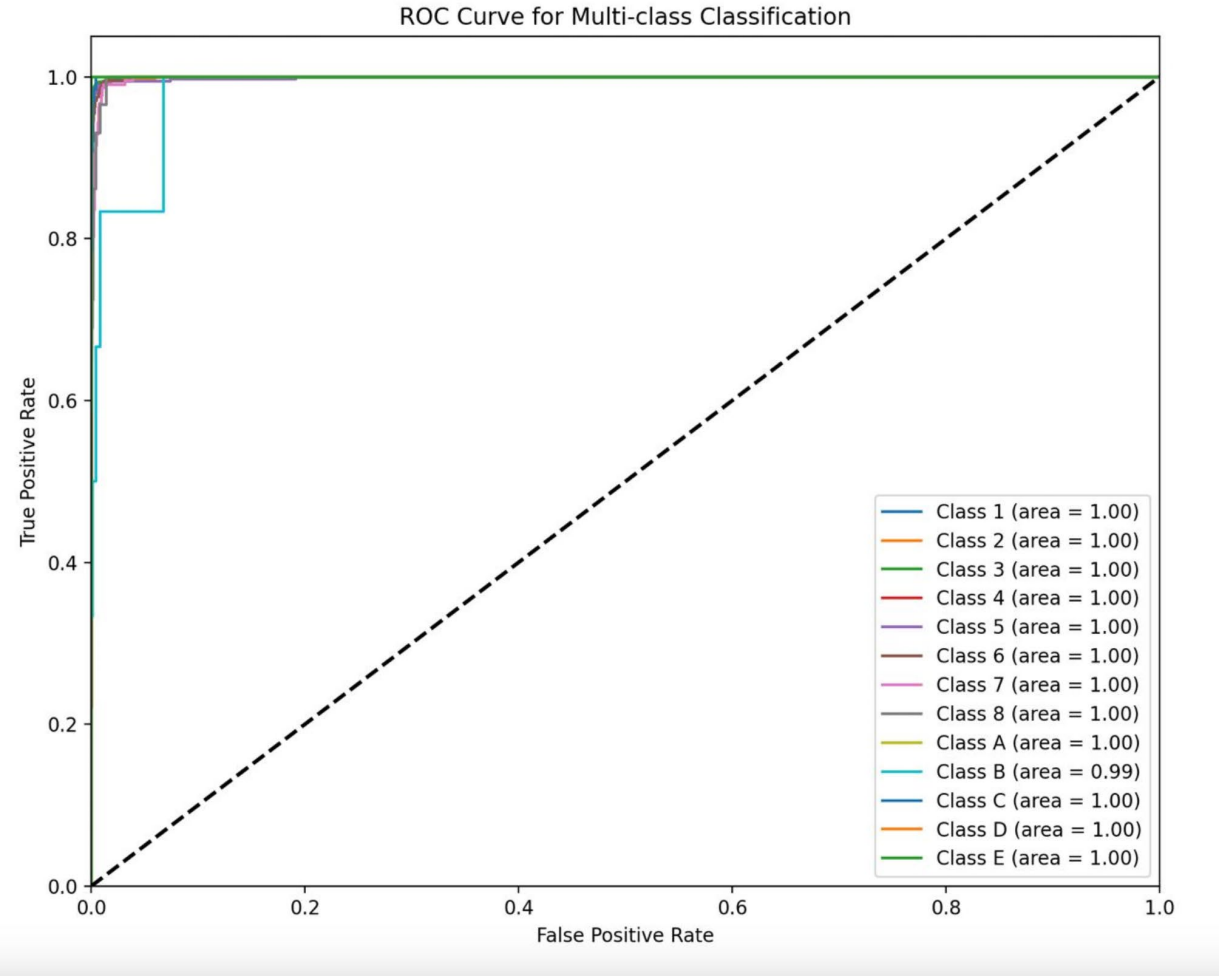
The ROC curve of the model, indicating very low false positive rate and high true positive rate in this study

**Fig. 5:**
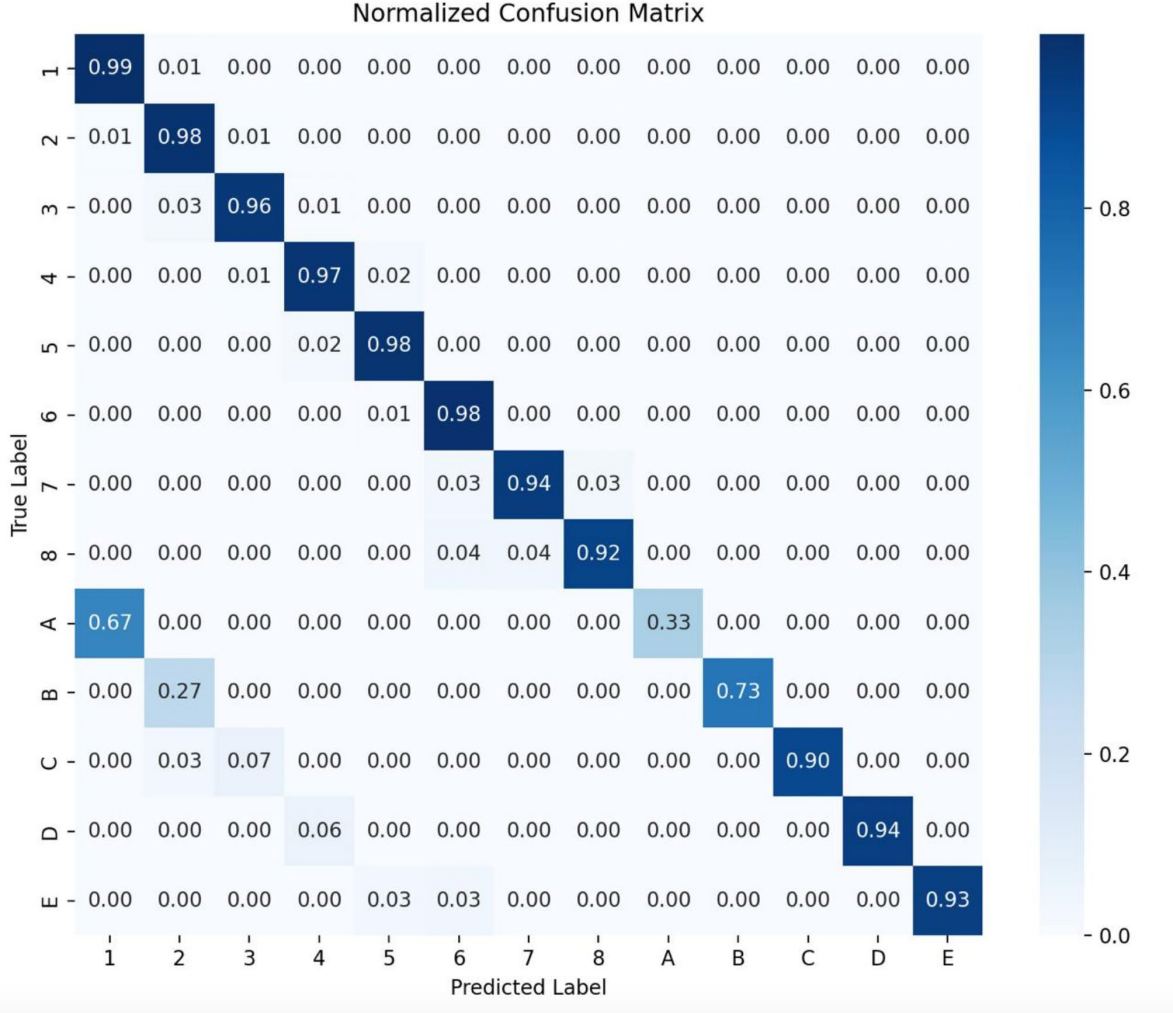
Confusion matrix of the model, showing the model’s performance for each tooth

**Fig. 6:**
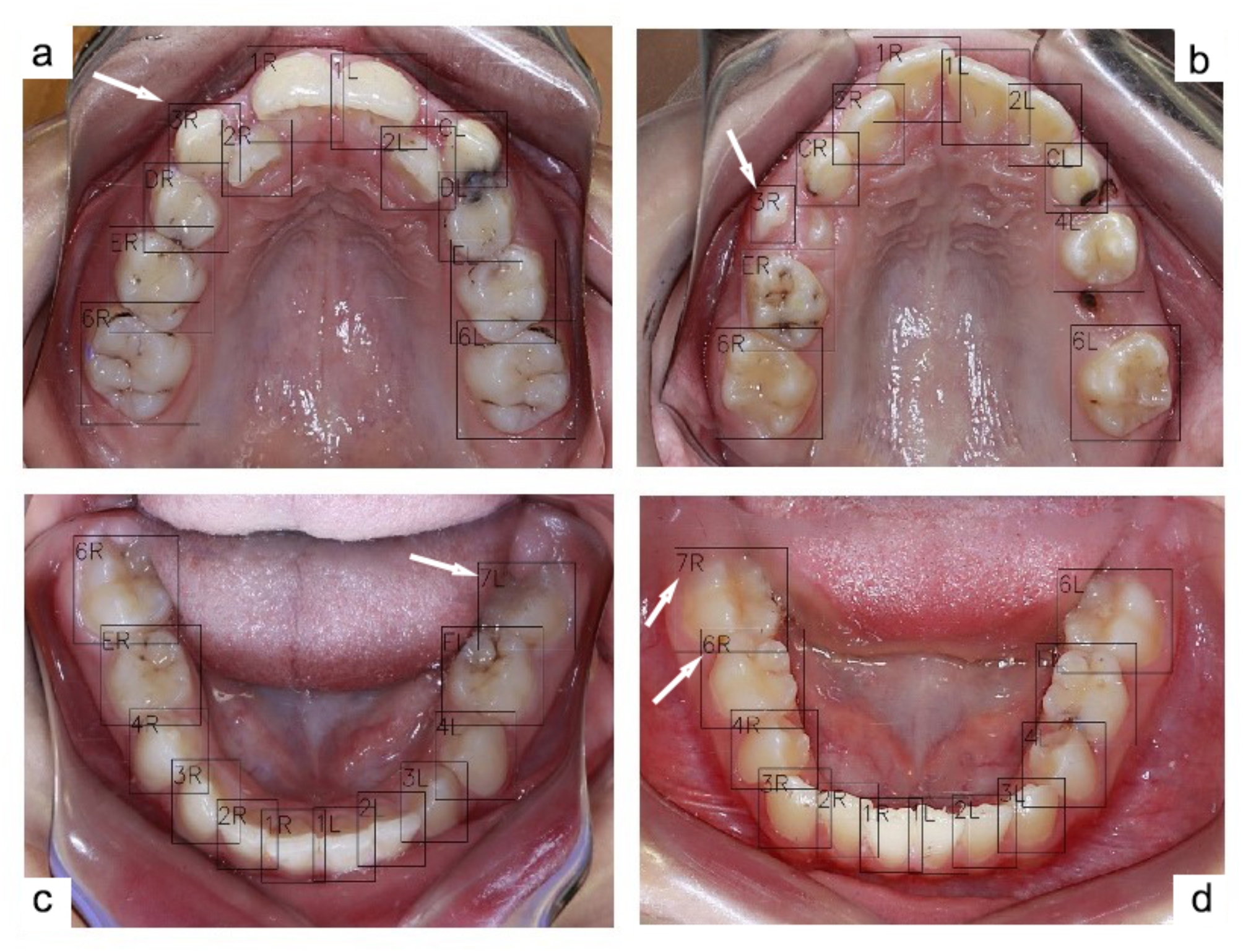
Examples of misclassifications during the test phase. White arrows point to incorrect numbering by the model, with some misclassified teeth (shown in images b and c) being partially erupted

As indicated in Fig. 4, The ROC curve demonstrates that all lines are clustered near the top left corner, indicating the model’s sensitivity and precision. Figure 5 presents our model’s confusion matrix, highlighting the most frequent decisions for each tooth. It reveals that primary canines were often confused with permanent canines, and primary molars were mostly misclassified as permanent first molars. Some frequent misclassifications of the model are presented in Fig. 6.

**Fig. 7:**
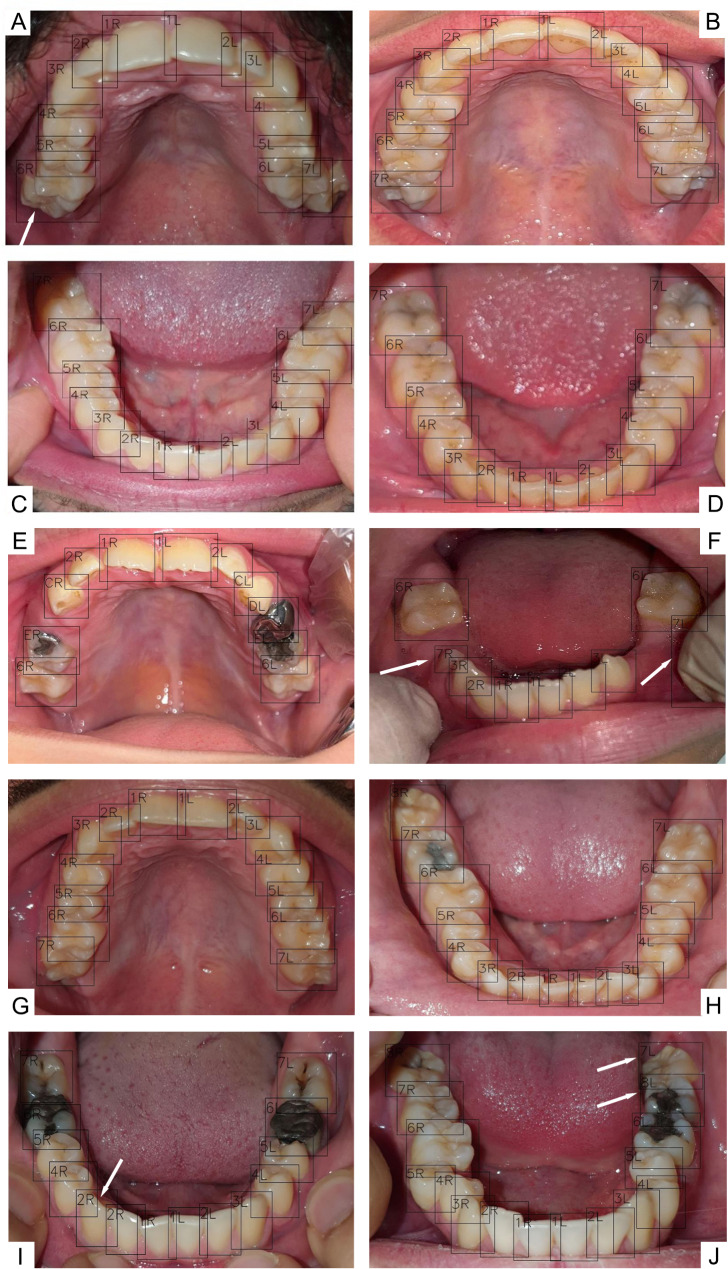
Examples of the model’s performance on photos taken with a smartphone. White arrows show the errors of the model

In the pilot test with a few smartphone photos, our model classified most teeth accurately, demonstrating its adaptability to non-standard images (Fig. 7).

## Discussion

The development of an AI-driven model for the detection and numbering of primary and permanent teeth on occlusal photographs marks a significant leap forward in the field of dental diagnostics, particularly in pediatric care. Dental health, especially in early childhood, plays a crucial role in overall health and development, influencing self-esteem, nutrition, and speech [19]. Traditional dental examinations require the physical presence of a dentist, which can be both time-consuming and resource-intensive. Access to timely and accurate dental assessments remains a challenge for many, particularly in rural or underserved areas which leads to untreated diseases and negative health consequences [20]. Our study represents progress toward addressing this issue, paving the way for future advancements in screening systems and tele-dentistry that will make timely disease diagnosis accessible and affordable for everyone.

The results of our study underscore both the strengths and limitations of our CNN model in the context of tooth detection and numbering from occlusal photographs. The model’s overall performance was robust, particularly with permanent teeth, but several challenges emerged that warrant further discussion. The model’s lower precision with primary teeth, especially the maxillary primary canines (upper C), were anticipated given the fact that most of the patients we had access to, were older than 12 years and primary teeth comprised a smaller portion of our dataset compared to permanent teeth. The maxillary primary canines, with an F1 score of 93.62%, were frequently misclassified due to their occlusal similarity to the permanent canines (Fig. 6). This challenge is exacerbated when primary teeth are underrepresented in the training data. If future datasets include a more balanced representation of primary teeth, particularly canines, we could expect substantial improvements in detection precision. The poor performance on primary incisors (teeth A and B), with F1 scores of 50% and 84.21%, respectively, is also due to the fact that our dataset predominantly consisted of images from children over six years old, where primary incisors are often exfoliated. The model had limited exposure to these teeth during training and is currently less reliable for detecting and numbering primary teeth in children under six years of age. The same reason contributed to the relatively lower precision scores for permanent third molars.

The high accuracy observed in permanent central incisors (99.6% F1 score) and most other permanent teeth demonstrate the model’s strong capabilities when sufficient data is available. The distinct occlusal features of these teeth, combined with their ample representation in the dataset, facilitated effective model training and reliable detection. However, the model’s difficulty in distinguishing between lower first and second molars is notable. The results for these teeth were slightly weaker, with an F1 score of 95.75% for mandibular first molars, largely due to the morphological similarities between these teeth. Furthermore, the model occasionally confused lower first molars with primary second molars (teeth E), a misclassification driven by their comparable occlusal patterns and the fact that tooth size, a critical differentiator of these teeth in the occlusal view, was not incorporated into the model’s analysis. Future iterations of the model could benefit from incorporating size as a feature to improve differentiation between these closely related teeth.

The model’s high sensitivity and area under curve (AUC) values can be attributed to the nature of the task. The primary task of the model was to detect and number teeth within an oral environment, where the contrast between the white teeth and the red soft tissue is highly distinct. This clear contrast significantly simplifies the detection process for the model, enabling it to reliably identify teeth even in cases where only a small part of the tooth, such as a cusp tip, is visible. The model was trained to recognize these partially erupted teeth, as they were labeled during the annotation process, resulting in extremely high detection sensitivity. While the model excels in detection of these partially erupted teeth, the challenge arises in the numbering phase, where misclassifications occur, leading to false positives. However, the false negative rate remains low. Figures 2 and 6, and 7 highlight cases where the model successfully detects partially erupted teeth, with some being correctly numbered and some being misclassified.

Our study builds on the foundation laid by previous research in the field of AI-driven dental diagnostics, pushing the boundaries of what is possible with current technology. Kurt-Bayrakdar et al. successfully utilized deep learning algorithms on oral photographs for tooth numbering and detection of periodontal conditions such as gingival hyperplasia and frenulum attachments [1]. Their model, based on the YOLOv5 architecture, demonstrated acceptable precision and F1 score (78% and 87%, respectively). However, their study was limited to images of patients older than 12 years and did not account for the unique challenges posed by the presence of primary teeth in mixed dentitions. Additionally, their dataset consisted of standard and high-quality images with similar angles, which may not accurately reflect the variability seen in the real world. Similarly, the study by Yoon et al. [5] focused on the detection of caries and tooth numbering using AI on intraoral photographs, achieving commendable results (an overall mAP of 88%). However, they also used high-quality images of permanent dentition and excluded those with the presence of saliva or food, scratches, orthodontic brackets, and primary teeth. A recent study by Nguyen et al. utilized YOLOv11 for tooth detection and numbering on intraoral images [15]. They also performed tooth segmentation using Light Segment Anything in High Quality (Light HQ-SAM). While their study did not report specific results for the numbering procedure, their segmentation model demonstrated high accuracy, achieving mean Dice similarity coefficients (DSC) of 0.983 for upper occlusal and 0.973 for lower occlusal images. Unlike our study, which focused exclusively on occlusal views, their model was trained on occlusal, frontal, and lateral perspectives. Similar to the previous two studies, Nguyen et al. also restricted their dataset to high-quality images of permanent dentition, explicitly excluding primary teeth, fixed appliances, blurriness, and poor lighting conditions. Our study differentiates itself by incorporating a more diverse dataset, including images taken under varying conditions, such as incorrect angles and the presence of orthodontic appliances, saliva, and artifacts like scratches. This approach not only enhances the model’s generalizability but also makes it more applicable to real-world situations where perfect imaging conditions cannot be guaranteed. Moreover, our model was specifically designed to handle mixed dentitions, a complex phase where both primary and permanent teeth coexist, which was not the primary focus of the previous studies [1, 5]. By addressing these gaps, our study provides a more comprehensive solution that is better suited for pediatric dental care. Additionally, this study achieved higher precision and F1 scores than both previous studies.

Many studies that use AI on intraoral photographs have focused primarily on caries detection. Some have even concluded that deep learning models outperform young dentists in diagnosing carious lesions, highlighting an opportunity for AI integration in both clinical and remote dental assessments [21, 22]. A systematic review conducted in 2022 identified 42 studies on automated caries detection, 12 of which were specifically conducted on intraoral photographs [23]. The review reported accuracy rates between 71% and 96% for AI-based caries detection in intraoral photographs. However, the critical limitation of these studies is their narrow focus on caries detection alone, without addressing the essential task of tooth numbering. This gap underscores the need for an AI-driven system capable of both tooth numbering and caries detection using intraoral images, a challenge that the current study aims to bridge.

Most previous research on automated tooth numbering has been conducted using dental radiographs rather than intraoral photographs. A recent systematic review examined deep learning-based tooth numbering on radiographs, identifying 29 studies [24]. Notably, only two of these studies included deciduous teeth, with the remaining research focusing solely on permanent dentition. This further highlights the necessity of extending AI models to mixed dentition, which our study attempts to address. The studies included in the review by Sadr et al. reported F1 scores ranging from 87 to 98% for tooth detection and numbering on radiographs [24]. Our model achieved an F1 score of 98.2% for permanent dentition on intraoral photographs, demonstrating that, despite the widespread reliance of the literature on radiographs for automated tooth numbering, AI can achieve comparable or even superior performance using intraoral images. Given that radiographic imaging is often inaccessible in rural or underserved regions and involves unnecessary radiation exposure, our findings suggest that intraoral photographs can serve as a viable and effective alternative for AI-driven dental assessments.

The backbone of our model’s success lies in its architecture, specifically the use of a convolutional neural network (CNN) framework with the YOLOv8 design. CNNs excel at processing large and complex images by extracting multiple features through layered filters, making them especially effective for image recognition and classification tasks [25]. The YOLO design, particularly in its latest iteration (YOLOv8), offers several advantages over traditional models. YOLO is designed for speed and accuracy, capable of processing images in real-time without compromising on performance [26]. This is particularly important in a clinical setting, where timely results are crucial. YOLOv8’s ability to detect multiple objects within an image, coupled with its efficiency in handling large datasets, makes it an excellent choice for dental image analysis [27].

One of the key innovations in our study is the inclusion of both standard and non-standard images for training the model. Traditional AI models in dentistry have relied heavily on standardized, high-quality images taken under controlled conditions. While this approach ensures high accuracy, it limits the model’s applicability in real-world scenarios. Our study deliberately included images with scratches, poor lighting, and incorrect angles, as well as those with orthodontic appliances to ensure that the model can still perform well, even when ideal conditions are not met. The results of our pilot test using images taken by smartphones, show this capability of our model (Fig. 7). Incorporating these non-standard images is vital for expanding the practical use of the model, particularly in tele-dentistry. Many families, especially those in remote or underserved regions, face challenges in accessing dental care due to distance, time, or financial constraints. By supporting the use of smartphone-captured images, our model offers a practical solution to bridge this gap. Parents and caregivers can easily take photos of their child’s teeth and receive a preliminary assessment, which not only conserves time and resources but also helps identify potential issues early, allowing for timely professional intervention before problems escalate.

This feature also holds substantial potential for epidemiological studies, especially in rural areas. Traditional studies measuring dental health indicators, like the decayed, missing, and filled teeth (DMFT) index, often overlook populations in remote regions due to limited accessibility. Our model can assist in overcoming this challenge by allowing health workers to gather accurate data from photos taken on-site, enhancing the completeness of public health data and enabling more targeted interventions. Although the initial pilot test on a limited number of smartphone-taken images yielded satisfactory results, it is expected that the model will encounter more challenges and make additional errors when processing these non-standard images. The inherent variability in image quality, such as incorrect focus and angle, blurry teeth, lighting conditions, and potential obstructions like saliva or food, often present in non-standard photos, could contribute to a higher rate of false positives and false negatives. Future research should apply the model to a larger dataset of smartphone-taken images. This will allow for a more comprehensive evaluation of its performance and provide a more accurate determination of its precision, sensitivity, and F1 score, ensuring the model’s effectiveness in non-standard images.

Despite its successes, our study faced certain limitations. The most significant challenge was the underrepresentation of specific tooth types in our dataset, particularly primary incisors, canines, and permanent third molars. This limitation resulted in lower accuracy for these tooth types. Expanding the dataset to include more images of primary incisors would enable the model to accurately detect and number teeth in children under six years old. Moreover, the model could be integrated with other systems and further developed to identify dental issues such as caries, missing or supernumerary teeth, and orthodontic concerns, which are crucial for early intervention.

While this study focuses on occlusal photographs, the growing adoption of 3D digital workflows in modern dentistry presents an opportunity for further advancements. Intraoral scanners provide highly detailed three-dimensional representations of dental structures. Future research could focus on developing deep learning models specifically trained on 3D intraoral scan data, enabling automated tooth detection and numbering in a fully digital environment. Integrating deep learning models with 3D intraoral scan data could enhance the precision of automated tooth detection and numbering and broaden the applicability of automated tooth analysis, particularly in cases where overlapping teeth or complex anatomical variations present challenges in 2D images.

## Conclusion

In conclusion, our study presents a significant advancement in the field of dental diagnostics through the development of an AI-based model for detecting and numbering primary and permanent teeth on occlusal photographs. The model’s high precision underscores its potential as a reliable tool for dental assessments, especially in pediatric care. By incorporating non-standard images, our model demonstrates robustness and applicability in real-world scenarios, offering a promising potential for tele-dentistry. This innovation not only facilitates early detection and intervention but also holds promise for enhancing epidemiological studies, particularly in underserved and rural populations.

## Data Availability

The datasets used during the current study are available from the corresponding author on reasonable request.

## Abbreviations

AI: Artificial intelligence
CNN: Convolutional neural network
YOLO: You only look once
IoU: Intersection over union
GIoU: Generalized intersection over union
VFL: Vertical federated learning
mAP: Mean average precision
ROC: Receiver operating characteristic
TP: True positive
FP: False positive
FN: False negative
AUC: Area under curve
DMFT: Decayed, missing, and filled teeth
